# Association Between Carotid Artery Perivascular Fat Density and Embolic Stroke of Undetermined Source

**DOI:** 10.3389/fneur.2021.765962

**Published:** 2022-02-18

**Authors:** Xiaohong Hu, Jianhui Chen, Huajun Fu, Yinjuan Chen, Daofeng Fan, Yangui Chen, Chaoxiong Shen

**Affiliations:** ^1^Neurology Department, Longyan First Affiliated Hospital of Fujian Medical University, Longyan, China; ^2^Emergency Department, Longyan First Affiliated Hospital of Fujian Medical University, Longyan, China

**Keywords:** carotid artery, ESUS, LAA strokes, perivascular fat density, computed tomography angiography

## Abstract

**Aim:**

This study aims to retrospectively evaluate the association between pericarotid inflammation and the presence of embolic stroke of undetermined source (ESUS).

**Methods:**

In total, 126 patients with ESUS and 118 patients with ischemic stroke from large artery atherosclerosis (LAA) were enrolled. All the patients underwent brain MRI and a neck CT angiography (CTA) examination. Reviewers were blinded to infarct location and stroke cause. Paired *t*-tests assessed within-subjects differences in mean Hounsfield units (HUs) in carotid perivascular fat between the cerebral infarction side and contralateral side for ESUS and LAA ischemic stroke cases. The unpaired Student's *t*-test was used to assess between-subjects differences in mean HUs between ESUS and LAA ischemic stroke cases.

**Results:**

In both the ESUS cases and LAA ischemic stroke cases, the pericarotid fat density around the carotid artery ipsilateral to the stroke significantly increased compared with contralateral stroke position in both the groups (ESUS cases −56.31 ± 18.70 vs. −67.31 ± 20.01, *p* = 0.000; LAA ischemic stroke cases −51.62 ± 19.95 vs. −64.58 ± 22.68, *p* = 0.000). However, there was no significant difference in ipsilateral and contralateral positions to infarct between ESUS cases and LAA ischemic stroke cases (ipsilateral to infarct −56.31 ± 18.70 vs. −51.62 ± 19.95, *p* = 0.059; contralateral to infarct −67.31 ± 20.01 vs. −64.58 ± 22.68, *p* = 0.320).

**Conclusion:**

We found increased density in the fat surrounding carotid artery ipsilateral to stroke compared with contralateral in ESUS, suggesting the presence of an inflammatory reaction that extends beyond the vessel lumen in patients with ESUS with a risk factor profile similar to LAA strokes.

## Introduction

Stroke is the second most common cause of death and imposes a substantial burden of permanent disability (1). Determining the cause of a stroke is important because etiological subtypes have varying recurrence risks and may require different treatments to best prevent recurrence. Current etiologic classification systems like the Trial of Org 10172 in Acute Stroke Treatment (TOAST) (2) assign stroke causes to one of four major categories: large artery atherosclerosis (LAA), cardiac embolism, small vessel occlusion, or other rare causes, and embolic stroke of undetermined source (ESUS). In about 17% of ischemic strokes, standard investigations fail to identify a cause and can only be classified as ESUS (3).

Recent findings suggested that mild-to-moderate carotid plaque involving <50% luminal narrowing may play an important pathogenic role despite not causing any hemodynamically significant stenosis, especially in ESUS (4–6). This suggests that vulnerable carotid plaques may rupture and cause downstream embolism even in the absence of significant luminal stenosis but with the high-risk non-stenosing plaque features. These findings suggest that some proportion of ESUS might be reclassified, if high-risk non-stenosing plaque features were considered.

Inflammation plays an important role in the stability of carotid artery atherosclerotic plaque and its complications (7). Recent studies have reported that pro-inflammatory cytokines released from the inflamed arterial wall diffuse to the perivascular adipose tissue induce lipolysis and inhibit local adipogenesis, also promoting perivascular edema. This changes the composition of perivascular fat around inflamed arteries that causes perivascular fat tissue with a higher water/lipid ratio and forces attenuation to rise to the less negative values [toward −30 Hounsfield units (HUs)] (8–11). However, similar to work in the coronary field, studies have provided evidence that perivascular fat density (PFD) on CT angiography (CTA) was increased around culprit lesions compared with non-culprit lesions in patients with acute cerebral infarction from LAA (12, 13). These studies suggest the feasibility of density variation in the carotid perivascular adipose tissue on CTA for the non-invasive assessment of carotid artery inflammation and risk stratification.

In this view, the aim of this study was to investigate the association between the carotid artery PFD on CTA and ESUS. This could have implications for the diagnostic strategy in patients with ESUS and allow a more complete cerebrovascular risk profile in this subgroup of patients.

## Methods

### Patients

This study was approved by the Human Subjects Institutional Review Board of Longyan First Affiliated Hospital of Fujian Medical University. We screened consecutive neck CTA examinations performed at our institution from January 1, 2017 to December 31, 2019 to identify patients meeting the following inclusion criteria: (1) ischemic stroke was confirmed based on both clinical findings and the results of brain MRI; (2) ischemic stroke in all the cases was due to LAA or ESUS, according to the TOAST classification system (2) and per the recently proposed definitions for ESUS (14); (3) all the patients have bilateral carotid plaque. Exclusion criteria included: (1) simultaneous bilateral anterior circulation events; (2) patients with simultaneous anterior and posterior circulation strokes; (3) patients with intracranial large artery stenosis; (4) patients without carotid plaque. We enrolled 126 patients admitted to our Stroke Unit (Longyan First Affiliated Hospital of Fujian Medical University) for ESUS which was defined according to criteria proposed by the Cryptogenic Stroke/ESUS International Working Group (15). We also evaluated a confirmation group composed of 118 patients with acute cerebral infarction from LAA ischemic stroke. Acute cerebral infarction was confirmed based on both clinical findings and the results of brain MRI. In order to further exclude cardioembolic stroke, relevant examinations were performed in all patients with ESUS. All the patients with ESUS underwent immune parameters (including autoimmune antibodies), anticardiolipin antibody, transthoracic echocardiography, 12-lead ECG, 24-h ECG, and transcranial Doppler microbubble test. All the patients had been monitored microemboly signals in the cerebral arteries for 1 h using a pulsed Doppler device when they were considered as ESUS.

### Baseline Information

Data on various risk factors were recorded including gender, age, smoking, and drinking information, history of diabetes mellitus, hypertension, heart disease, chronic obstructive pulmonary disease (COPD), triglycerides (TGs), total cholesterol (TC), high-density lipoprotein cholesterol (HDL-C), low-density lipoprotein cholesterol (LDL-C), C-reactive protein (CRP), fibrinogen, and levels of D-dimer.

### Imaging Technique

Computed tomography angiography of the carotid arteries was performed on one of several scanners at our imaging sites including Light Speed Pro-16 or HD-750 (GE Healthcare, Milwaukee, Wisconsin, USA). The coverage was from the aortic arch to the carotid siphon using helical scanning mode. Collimation was performed at 0.625 mm, with a peak kilo voltage of 120 and automilliampere, and a rotation time of 0.5 s. An angiographic phase was obtained with the administration of 50–70 mL of non-ionic iodinated contrast (iohexol, Omnipaque, GE Healthcare, Milwaukee, Wisconsin, USA) via an 18-gauge peripheral intravenous catheter at 4–5 ml/s by using a power injector and SmartPrep software (GE Healthcare, Milwaukee, Wisconsin, USA) region of interest (ROI) on the aortic arch. The examination was performed by physicians in the Department of Radiology.

### Imaging Data Analysis

**Figure 1:**
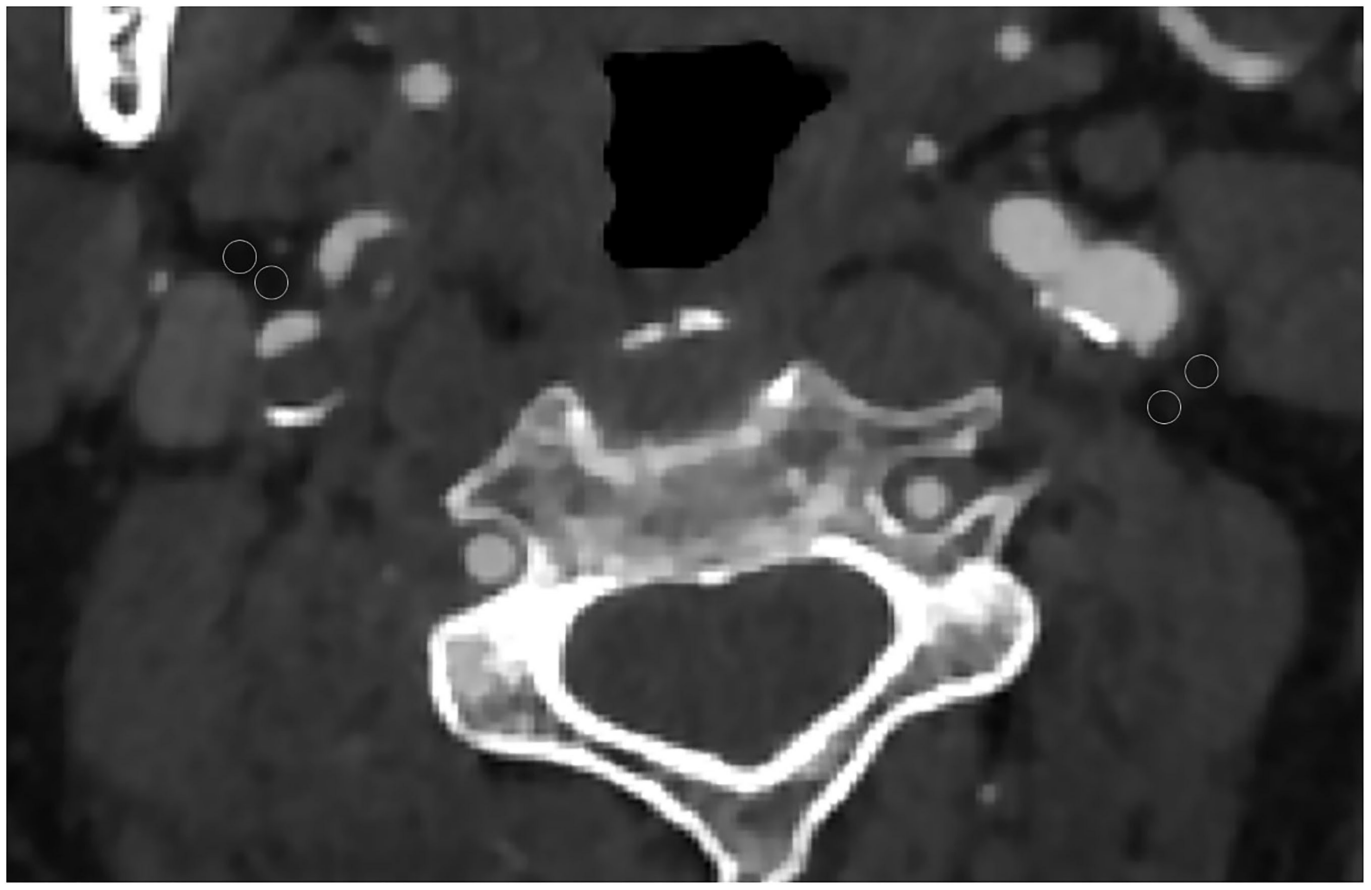
Axial CT angiography image of a 65-year-old male patient. Two regions of interest (ROIs) were placed in the perivascular fat for measured perivascular fat density. In this case, the 2 left ROIs were −60 and −68 and the 2 right ROIs were −62 and −73.

Each CTA was reviewed by two neuroradiologists who were blinded to all the clinical information including the location of cerebral infarction. The reviewer was not permitted to view additional neuroimaging other than the neck CTA. The pixels corresponding to adipose tissue were identified, and in the perivascular fat present on the same axial slice showing the maximal North American Symptomatic Carotid Endarterectomy Trial (16) defined atherosclerotic plaque or carotid artery stenosis placed 2 ROIs (each 2.5 mm^2^). The site of ROI placement was determined at the maximum stenosis site, location of the carotid atherosclerotic plaque, and location of the perivascular fat pads (Figure 1). ROIs were drawn carefully to only include detectable fat density (visually dark and confirmed by negative HUs). Care was taken to exclude the carotid artery wall or surrounding soft-tissue structures, with ROIs placed at least 1 mm from the outer margin of the carotid artery wall.

### Statistical Analysis

Statistical analyses were performed by using the IBM SPSS Statistics version 20.0 software (IBM Corporation, Armonk, New York, USA). The means and SDs were calculated for normally distributed continuous variables and percentages were calculated for categorical variables. Differences in categorical variables were analyzed by the chi-squared test and continuous variables were compared using the Student's *t*-test. We performed both between-subjects and within-subject analyses. First, we compared the PFD within-subject, comparing the cerebral infarction side to the contralateral side, respectively, in ESUS cases and LAA ischemic stroke cases. Paired *t*-tests were used to assess the difference in perivascular fat densities between the cerebral infarction side and the contralateral side of the same patient. Second, we used the unpaired Student's *t*-test to perform a between-subjects comparison of PFD around the carotid artery for patients with ESUS cases compared with LAA ischemic stroke cases. All the fat density measurements are reported in HU.

## Results

### Cohort Characteristics

A total of 244 participants were enrolled in this study including 126 ESUS cases and 118 LAA ischemic stroke cases. The demographic and clinical characteristics of ESUS cases and LAA ischemic stroke cases are shown in Table 1. The mean age, number of smokers, drinkers, rates of diabetes mellitus, hypertension, coronary heart disease, COPD, levels of TGs, TC, HDL-C, LDL-C, CRP, fibrinogen, and levels of D-dimer were not significantly different between ESUS cases and LAA ischemic stroke cases (*p* > 0.05). There was a significantly higher proportion of males in the LAA ischemic stroke cases (*p* < 0.05).

**Table 1:** Baseline characteristics.

**Characteristics**	**ESUS cases *n* = 126**	**LAA ischemic stroke cases *n* = 118**	***P* value**
Male, *N* (%)	65.87	80.51	0.010
Mean age, *N* (years)	68.11 ± 10.33	68.82 ± 11.20	0.606
Smoke, *N* (%)	46.82	50.85	0.530
Alcohol consumption, *N* (%)	38.10	38.14	0.995
Diabetes mellitus, *N* (%)	35.71	27.12	0.149
Hypertension, *N* (%)	69.84	66.95	0.627
Coronary artery disease, *N* (%)	7.94	14.41	0.107
COPD, *N* (%)	22.22	19.49	0.600
TG (mmol/L)	1.96 ± 1.61	1.61 ± 1.11	0.051
TC (mmol/L)	4.95 ± 1.16	4.68 ± 1.22	0.081
HDL-C (mmol/L)	1.29 ± 0.35	1.33 ± 0.51	0.532
LDL-C (mmol/L)	3.25 ± 1.05	3.08 ± 1.08	0.227
C-reactive protein, mg/L	3.22 ± 3.78	3.40 ± 3.84	0.719
Fibrinogen, μmol/L	2.91 ± 0.82	3.14 ± 1.11	0.075
D-dimer, mg/L	0.77 ± 1.04	1.08 ± 1.41	0.051

*ESUS, embolic stroke of undetermined source; LAA, large artery atherosclerosis; COPD, chronic obstructive pulmonary disease; TGs, triglycerides; TC, total cholesterol; HDL-C, high-density lipoprotein cholesterol; LDL-C, low-density lipoprotein cholesterol*.

### Comparison of PFD Between Subjects and Within Subjects

In the within-subject analysis of the carotid artery ipsilateral to the stroke vs. contralateral in both the ESUS cases and LAA ischemic stroke cases (Table 2), we found significantly increased PFD around the carotid artery ipsilateral to the stroke vs. the contralateral in both the groups (ESUS cases −56.31 ± 18.70 vs. −67.31 ± 20.01, *p* = 0.000; LAA ischemic stroke cases −51.62 ± 19.95 vs. −64.58 ± 22.68, *p* = 0.000).

**Table 2:** Perivascular fat density on CT angiography (measured in HU).

**Characteristic**	**Ipsilateral to infarct**	**Contralateral to infarct**	***P* value**
ESUS cases	−56.31 ± 18.70	−67.31 ± 20.01	0.000
LAA ischemic stroke cases	−51.62 ± 19.95	−64.58 ± 22.68	0.000
*P* value	0.059	0.320	

*ESUS, embolic stroke of undetermined source; LAA, large artery atherosclerosis; HUs, Hounsfield units*.

We also performed a between-subjects analysis comparing PFD differences between the patients with ESUS cases and LAA ischemic stroke cases both ipsilateral and contralateral to infarct (Table 2). There was no significant difference both ipsilateral and contralateral to infarct between ESUS cases and LAA ischemic stroke cases (ipsilateral to infarct −56.31 ± 18.70 vs. −51.62 ± 19.95, *p* = 0.059; contralateral to infarct −67.31 ± 20.01 vs. −64.58 ± 22.68, *p* = 0.320).

## Discussion

Untangling embolic sources contributing to ESUS is important to better inform targeted secondary prevention. However, the exact causes of ESUS remain unknown and the most effective antithrombotic therapy remains a question. Recent studies have shown that nearly one-third of the patients with ESUS had evidence of increased P-wave terminal force in lead V1 and it was inversely associated with artery-to-artery and paradoxical potential sources of stroke (17). These findings indicated that left atrial cardiopathy could be involved in the pathogenesis of ESUS. Simona Lattanzi et al. (18) found that the ESUS population can be identified to distinct clinical phenotypes including the group linked infarct of anterior vascular territory, ipsilateral substenotic vulnerable carotid atherosclerosis, smoking status, and dyslipidemia. Emerging evidence suggests that non-stenosing LAA is one of the most important underlying mechanisms of ESUS (19–21). In the ESUS Global Registry, 79% of patients had non-stenotic plaques in the cervical carotid arteries (22). Previous studies have shown that inflammation in vulnerable atherosclerotic plaques is closely related to ipsilateral cerebral infarction (23) and inflammation associated with culprit carotid plaques extends beyond the vessel lumen and can be identified via a simple CTA imaging method (13).

In this study, we examined the association between carotid artery PFD and ischemic stroke. We found the ipsilateral side to the stroke had a higher carotid artery PFD compared to the contralateral side in both the ESUS cases and LAA ischemic stroke cases. This is similar to the findings of Hediyeh Baradaran et al. (13), showing that patients with unilateral internal carotid artery (ICA) stenosis, ≥50–99% of the fat surrounding ICAs ipsilateral to stroke or transient ischemic attack had higher mean pericarotid fat density compared with asymptomatic ICAs. Our findings overall suggest that there is increased inflammation in the fat surrounding the carotid artery ipsilateral to the infarct in both the ESUS cases and LAA ischemic stroke cases. When comparing the PFD between the patient ESUS cases and LAA ischemic stroke cases, there was no significant difference between both sides ipsilateral and contralateral to infarct. Previous studies have shown that the presence of significant artery atherosclerosis is associated with an increased perivascular fat inflammation (24). Our findings also suggest that in ESUS, the perivascular fat of the carotid artery on the infarct side has an obvious inflammatory reaction. The infarct side is the same as LAA ischemic stroke cases and these inflammatory changes in perivascular fat can be evaluated by differences in the fat density as measured by HU on CT.

This study has limitations that should be considered. First, the recruitment was monocentric with a small sample size. Second, in the ESUS group, although efforts have been made to exclude patients with cardiogenic stroke, the possibility of cardiogenic stroke cannot be completely excluded because of paroxysmal arrhythmia in some patients. Third, the lack of a control group of patients with other etiologies including cardioembolic and lacunar stroke is a potential limitation of this study. In addition, the occurrence of ischemic stroke is partly influenced by environmental and genetic factors including familial, racial, and ethnic background, so the genetic background among ischemic stroke subtypes cannot be ruled out. In this study, individuals included in the analysis were all Chinese people and, therefore, the results may not be generalized to the other ethnic groups.

## Conclusion

From this study, we found an increased density in the fat surrounding carotid artery ipsilateral to stroke compared with contralateral in ESUS, suggesting the presence of an inflammatory reaction that extends beyond the vessel lumen in patients with ESUS with a risk factor profile similar to LAA strokes. For a long time, researchers have been using the measured stenosis percentage as the main standard to define high-risk carotid atherosclerotic diseases, often ignoring the unstable carotid plaque as an important source of cerebral embolism. Our findings may have implications for antithrombotic management and future research in patients with ESUS.

## Data Availability Statement

The raw data supporting the conclusions of this article will be made available by the authors, without undue reservation.

## Ethics Statement

The studies involving human participants were reviewed and approved by the Human Subjects Institutional Review Board of Longyan First Hospital Affiliated to Fujian Medical University. The patients/participants provided their written informed consent to participate in this study.

## Author Contributions

XH and CS designed and performed the experiments and wrote the manuscript. JC, HF, YiC, DF, and YaC collected and analyzed the data. All authors have read and approved the final version of the manuscript.

## Funding

This study was sponsored by the Longyan City Science and Technology Plan Project (Grant No. 2020LYF17030).

## Conflict of Interest

The authors declare that the research was conducted in the absence of any commercial or financial relationships that could be construed as a potential conflict of interest.

## Publisher's Note

All claims expressed in this article are solely those of the authors and do not necessarily represent those of their affiliated organizations, or those of the publisher, the editors and the reviewers. Any product that may be evaluated in this article, or claim that may be made by its manufacturer, is not guaranteed or endorsed by the publisher.
